# Metabolomic Links between Sugar-Sweetened Beverage Intake and Obesity

**DOI:** 10.1155/2020/7154738

**Published:** 2020-04-13

**Authors:** Bingjie Zhou, Reiko Ichikawa, Laurence D. Parnell, Sabrina E. Noel, Xiyuan Zhang, Shilpa N. Bhupathiraju, Caren E. Smith, Katherine L. Tucker, Jose M. Ordovas, Chao-Qiang Lai

**Affiliations:** ^1^Nutrition and Genomics Laboratory, JM-USDA Human Nutrition Research Center on Aging at Tufts University, Boston, MA, USA; ^2^Institute for Innovation, Ajinomoto Co., Inc., Kawasaki, Japan; ^3^USDA Agricultural Research Service, Nutrition and Genomics Laboratory, JM-USDA Human Nutrition Research Center on Aging at Tufts University, Boston, MA, USA; ^4^Department of Biomedical and Nutritional Sciences, University of Massachusetts Lowell, Lowell, MA, USA; ^5^Channing Division of Network Medicine, Harvard Medical School and Brigham and Women's Hospital, Boston, MA, USA; ^6^Department of Nutrition, Harvard T. H. Chan School of Public Health, Harvard University, Boston, MA, USA; ^7^IMDEA Food Institute, CEI UAM-CSIC, Madrid, Spain; ^8^Centro Nacional de Investigaciones Cardiovasculares (CNIC), Madrid, Spain

## Abstract

**Background:**

Sugar-sweetened beverage (SSB) consumption is highly associated with obesity, but the metabolic mechanism underlying this correlation is not understood.

**Objective:**

Our objective was to examine metabolomic links between SSB intake and obesity to understand metabolic mechanisms.

**Design:**

We examined the association of plasma metabolomic profiles with SSB intake and obesity risk in 781 participants, aged 45–75 y, in the Boston Puerto Rican Health Study (BPRHS) using generalized linear models, controlling for potential confounding factors. Based on identified metabolites, we conducted pathway enrichment analysis to identify potential metabolic pathways that link SSB intake and obesity risk. Variants in genes encoding enzymes known to function in identified metabolic pathways were examined for their interactions with SSB intake on obesity.

**Results:**

SSB intake was correlated with BMI (*β* = 0.607, *P*=0.045). Among 526 measured metabolites, 86 showed a significant correlation with SSB intake and 148 with BMI (*P* ≤ 0.05); 28 were correlated with both SSB intake and BMI (*P* ≤ 0.05). Pathway enrichment analysis identified the phosphatidylcholine and lysophospholipid pathways as linking SSB intake to obesity, after correction for multiple testing. Furthermore, 8 of 10 genes functioning in these two pathways showed strong interaction with SSB intake on BMI. Our results further identified participants who may exhibit an increased risk of obesity when consuming SSB.

**Conclusions:**

We identified two key metabolic pathways that link SSB intake to obesity, revealing the potential of phosphatidylcholine and lysophospholipid to modulate how SSB intake can increase obesity risk. The interaction between genetic variants related to these pathways and SSB intake on obesity further supports the mechanism.

## 1. Introduction

Obesity has become a major health burden in the US and worldwide [1, 2]. The increase in obesity is highly associated with modern lifestyle habits, including dietary intake, and their interactions with genetic predisposition [3–6]. Sugar-sweetened beverage (SSB) consumption is recognized as a major source of added sugar and energy in the US population [7, 8] and a key single dietary factor associated with the obesity epidemic [4, 9, 10] as well other metabolic diseases, such as type 2 diabetes and nonalcoholic fatty liver disease [11, 12]. Therefore, there is an urgent need to understand the mechanisms underlying the association between SSB consumption, obesity, and related diseases.

Metabolomics is a powerful tool to identify and measure a wide range of metabolites in biological samples, which can define metabolic profiles and link dietary intake and nutrient metabolism [13, 14]. Metabolomic profiles can also characterize metabolic states and interactions between diet and genes [15]. Metabolites measured in plasma have been associated with obesity and other metabolic diseases [16, 17]. Hence, metabolites can be used as biomarkers for the prediction of the progression of metabolic diseases.

Among the most informative indicators of metabolic status are the phospholipids, which function as the most crucial lipid component of the cell membrane. Alteration in the ratio of phosphatidylcholine (PC) and phosphatidylethanolamine (PE) in several tissues has been associated with metabolic disease [18]. Plasma phospholipids measured by metabolomic analysis are recognized as metabolic signatures linked to obesity, insulin resistance, and inflammation [19]. Impairment in lysophospholipid metabolism is correlated with obesity and desensitization to *n* − 3 polyunsaturated fatty acid intake [20]. Decreased lysophospholipids are associated with obesity, whereas increased phospholipids indicate risk of metabolic disease [21].

We hypothesized that SSB consumption dysregulates metabolic pathways and that this dysregulation, in turn, leads to increased risk of obesity. We first examined the association between plasma metabolites, SSB intake, and obesity among participants from the Boston Puerto Rican Health Study (BPRHS), which has a high prevalence of obesity, and then identified those metabolic pathways enriched by such associations. To test if SSB consumption indeed regulates the identified pathways, we selected a set of genes related to those pathways and examined whether there are interactions between SSB intake and genotypes influencing BMI.

## 2. Materials and Methods

### 2.1. Boston Puerto Rican Health Study (BPRHS)

The BPRHS is a longitudinal cohort study designed to investigate the relationship between stress, nutrition, and health outcomes, such as depressive symptomatology, cognitive impairment, functional limitations, and metabolic diseases in Puerto Ricans [22, 23]. Of the 1504 recruited participants aged 45–75 y from the greater Boston area who completed the baseline interview and clinical exams, 1311 had complete dietary, clinical, and biochemistry measures. Plasma samples from 817 participants were sent to Metabolon Inc. (Morrisville, NC, USA) for metabolomic analysis. In this study, metabolome profiles were available for 781 participants who had complete sugar-sweetened beverage intake data and all the clinical measures (see [Supplementary-material supplementary-material-1]).

### 2.2. Assessment of Sugar-Sweetened Beverage (SSB) Intake

Dietary intake was assessed using a food frequency questionnaire that was designed and validated for use in the BPRHS [24]. Regular and diet soft drinks, fruit drinks, sweetened energy drinks, and tea intake were assessed together as sweetened beverages. These include [1] regular cola and caffeine-free cola; [2] carbonated drinks with added sugar; [3] juice or flavored drink, fruit-flavored nectar (peach, pear, mango, lemonade), and fruit punch, but excluding 100% fruit juice; [3] other flavored drinks with vitamins and added sugar; and [4] purchased presweetened ready-to-drink tea. Total SSB intake was calculated and converted to servings per day.

### 2.3. Genotyping

Genome-wide genotyping was conducted using Affymetrix's Axiom Genome-Wide LAT Array, which was designed especially for Hispanic populations, and contains probe sets to genotype 817,810 SNPs. Genome-wide genotypes were called and quality control was performed using Genotype Console (GTC), Affymetrix® Power Tools (APT), and R, following standard protocols/best practices provided by the vendor. Based on the criteria of SNPolisher [25], 804,947 SNPs passed general QC. Among them, 717,275 autosomal SNPs met the following criteria: call rate ≥97%, minor allele frequency (MAF) ≥1%, and *P* value of Hardy–Weinberg Equilibrium (HWE) ≥10^−6^. To estimate population structure, 50,704 SNPs were selected based on the following criteria: call rate >97%, MAF ≥ 5%, pair-wise linkage disequilibrium *R* square ≤0.1, and *P* value of Hardy–Weinberg Equilibrium (HWE) ≥10^−6^. Using principal components analysis implemented in SVS (Golden Helix Inc.), one eigenvalue was selected to represent the population structure based on the scree plot. This principal component factor was included in all regression models to adjust for population structure. For this study, the genotypes of 83 SNPs in 10 genes of the identified pathway were available and used for examining gene-diet interaction with SSB intake on obesity.

### 2.4. Metabolomic Profiling

Metabolomic profiling was performed on plasma samples collected at baseline from participants in the BPRHS by Metabolon Inc. [26]. In short, plasma samples were shipped on dry ice to Metabolon and stored at −80°C before analysis. After proteins were removed with methanol, metabolomic analysis was performed using ultrahigh-performance liquid chromatography-tandem mass spectroscopy. With reference to a library of over 4500 purified standards for retention time/index, mass-to-charge ratio, and chromatographic data, individual metabolites were identified and quantified by estimating the AUC of the peaks [26]. After standardization across all the samples, 526 targeted metabolites were identified; among them, 432 were assigned to 54 metabolic pathways based on the presence of at least three metabolites within each pathway.

### 2.5. Statistical Analysis

All statistical analyses were conducted using SAS 9.4 (SAS Inc.), R 3.5.1, or SVS 8.8 (Golden Helix Inc.).

### 2.6. Metabolic Signature Analysis

Linear regression analyses were conducted to identify metabolites that were associated with both BMI and SSB intake. For the association between BMI and SSB intake, a linear regression model was used, with BMI as the dependent variable and SSB intake as the independent variable, adjusting for sex, age, physical activity, education, smoking, alcohol use, and medication for diabetes and hypertension.

To identify metabolic signatures of SSB intake in relation to obesity, we first performed a linear regression analysis with BMI as the dependent variable and the plasma concentration of each metabolite as an independent variable, controlling for sex, age, physical activity, education, smoking, alcohol use, and total energy intake. Second, a linear regression model was applied with each natural log-transformed metabolite concentration as a dependent variable and SSB intake as an independent variable, controlling for the same set of covariates as above. Metabolites that were significant (*P* ≤ 0.05) for both models were identified as metabolic signatures of SSB intake in relation to BMI. For the purpose of identifying the metabolic link between SSB intake and obesity, instead of correcting for multiple testing in each step, we selected those metabolites that were associated nominally (*P* ≤ 0.05) with both SSB intake and BMI as SSB-obesity metabolomic signatures. In essence, this step reduces the false positive rate, which serves as a correction for multiple testing. Furthermore, we corrected for multiple testing when conducting metabolic pathway enrichment analysis (see below).

### 2.7. Metabolic Pathway Enrichment Analysis

All detected metabolites were organized into metabolic pathways based on the annotation database of Metabolon Inc., and pathways that contained three or more metabolites were included in this analysis. The proportion of significant metabolites in each pathway was calculated and generated a *Z* score for each pathway. $Z\;\text{score}=\left[r-n\left(R/N\right)\right]/\sqrt{\left\{n\left(R/N\right)\left[1-\left(R/N\right)\right]\left[1-\left(n-1\right)/\left(N-1\right)\right]\right\}}$, where *N* is the total number of metabolites assigned to this metabolic pathway, *n* is the total number of metabolites measured in a specific pathway, *R* is the total number of metabolites that were significantly identified in the metabolic signature analysis as described above, and *r* is the number of *R* metabolites that were identified as significant in a specific pathway. *P* values were derived from *Z* scores assuming a normal distribution and were two-sided.

We considered the whole process of metabolic enrichment analysis as one test. Hence, the *P* value for each metabolite that was associated with SSB intake or BMI was designated as the strength of the correlation, and as such the *P* values were not corrected for multiple testing before metabolic enrichment analysis. Correction for multiple tests was done only once at the endpoint of the pathway enrichment analysis. The Bonferroni test was applied to correct for multiple testing in this step.

### 2.8. Interaction between Genetic Variants at Genes of Metabolic Pathways and SSB Intake on Obesity

We further examined interactions between genetic variants at the 10 genes that were selected from the identified metabolic pathways linking SSB intake with obesity. Eighty-three SNPs from these 10 genes in the PC/PE biosynthetic pathwa*y* were examined for GxE interaction between genotype and SSB intake using a linear regression model, with BMI as the dependent variable, and SSB intake and SNPs and their interaction as predictors, controlling for potential confounding factors: sex, age, physical activity score, smoking, alcohol use, and population structure. When the minor allele frequency (MAF) for a given SNP was <0.10, a dominant genetic model was applied. Otherwise, an additive model was used. False discovery rate (FDR) was used to correct for multiple testing.

## 3. Results

### 3.1. General Characteristics of the Study Population

A total of 781 participants had complete dietary, metabolome, and genotype data. The mean age of this subpopulation was 57.3 y, with 71% being women. This population had a high prevalence of obesity (mean BMI = 32.1), type 2 diabetes (39.3%), and hypertension (70%). Mean SSB intake, excluding 100% fruit juice, was 0.49 servings per day (95% CI = 0.43–0.55), and the mean total energy intake was 2113 kcal (95% CI = 2058–2167). Compared to all the participants (*n* = 1311), participants with metabolome profile (*n* = 781) showed no significant differences in any of the characteristics listed (Table 1).

### 3.2. SSB Intake Associated with Obesity

Adjusting for sex, age, smoking, drinking, medications for diabetes and hypertension, and physical activity, we identified a positive association between SSB intake and BMI (beta = 0.607, *P* value = 0.045, *n* = 781) in participants with metabolome profile. For all participants, the association between SSB intake and BMI was slightly weaker (beta = 0.420, *P* value = 0.070, *n* = 1311).

### 3.3. Association between Metabolites with SSB Intake and BMI

To identify metabolite signatures related to SSB intake, we examined the association between SSB intakes and metabolome profiles. Among 526 targeted metabolites, we found 86 metabolites nominally associated with SSB intakes at *P* ≤ 0.05 adjusted for sex, age, drinking, smoking, education, physical activities, and total energy intake ([Supplementary-material supplementary-material-1]).

We conducted an association study between metabolites and BMI. Using a similar regression model to that described above, 148 metabolites were nominally correlated with BMI at *P* value ≤0.05 while adjusting for the same covariates ([Supplementary-material supplementary-material-1]). In total, we found that 28 of 432 metabolites were commonly associated with both SSB intake and BMI, and we nominated them for SSB-obesity metabolomic signature (Table 2). For the purpose of identifying the metabolic links between SSB intake and obesity, instead of correcting for multiple testing in each step, we selected the metabolites that were associated nominally (*P* ≤ 0.05) with both SSB intake and BMI as SSB-obesity metabolomic signatures.

### 3.4. Pathway Enrichment Analysis

To identify metabolic signatures of SSB intake, we conducted enrichment analysis based on 86 metabolites that were nominally associated with SSB intake. Of 526 metabolites, 432 were related to 53 metabolic pathways with at least 3 metabolites measured. Of these 53 metabolic pathways, we observed four that were overrepresented by metabolites associated with SSB intake ([Supplementary-material supplementary-material-1]): phosphatidylcholine (PC), phosphatidylinositol (PI), lysophospholipid (LPL), and acyl choline metabolism (AC), after correction for multiple testing (*P* ≤ 0.05/53 = 0.001). A complementary analysis was carried out to identify a metabolic signature of BMI using a similar enrichment analysis, employing the 148 metabolites that were associated with BMI ([Supplementary-material supplementary-material-1]). This analysis identified long-chain fatty acids as significant (*P* ≤ 0.001) after correcting for multiple testing.

To identify metabolic pathways that potentially link SSB intake to BMI, we then performed an enrichment analysis to identify pathways that were overrepresented by 28 SSB-obesity metabolomic signatures associated with both SSB intake and BMI ([Supplementary-material supplementary-material-1]). Two pathways, LPL (*P*=5.65*E* − 06) and PC (*P*=1.30*E* − 04) metabolism, significantly stood out, after correction for multiple testing. LPL and PC metabolism are a key part of biosynthetic pathways of PC and phosphatidylethanolamine (PE) ([Supplementary-material supplementary-material-1]). Six SSB-BMI metabolomic signatures with 19 metabolites were overrepresented in the LPL pathway, and four metabolites with 6 metabolites were enriched in the PC pathway (Table 3). Strikingly, in both identified pathways, all 10 SSB-BMI metabolites were positively correlated with SSB intake but negatively associated with BMI.

### 3.5. Interaction between Genes of the PC/PE Biosynthetic Pathways and SSB Intake on BMI

**Figure 1:**
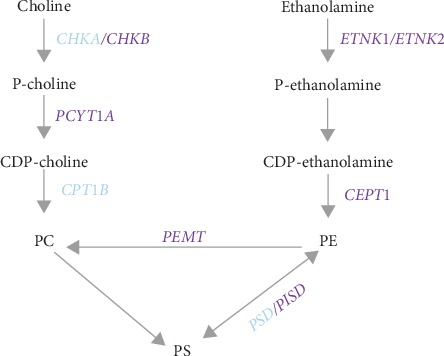
PC/PE biosynthesis pathway and related genes. Those genes that show significant interactions with SSB intake on BMI are indicated in purple. *CHKA* and *CHKB*: choline kinase alpha and beta; *ETNK1* and *2*: ethanolamine kinase 1 and 2; *CEPT1*: choline/ethanolamine phosphotransferase 1; *CPT1B*: carnitine palmitoyltransferase 1B; *PCYT1A* and *PCYT1B*: *CTP*: phosphoethanolamine cytidylyltransferase alpha and beta; *PEMT*: phosphatidylethanolamine N-methyltransferase; PC: phosphatidylcholine; PE: phosphatidylethanolamine; *PISD*: phosphatidylserine decarboxylase; *PSD*: pleckstrin and Sec7 domain containing; P-: phospho; CDP-: cytidine diphosphate.

**Figure 2:**
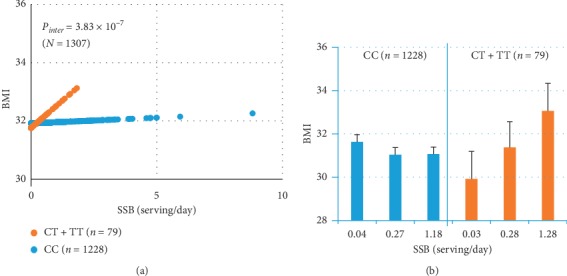
Interaction between *PEMT*-rs72828480 and SSB intake on BMI. *P* values and predicted BMI were calculated while adjusted for sex, age, smoking, alcohol use, physical activity, population structure, and total energy. (a) Predicted BMI was plotted against SSB intake (serving/day) according to PEMT-rs7282480 genotype. Orange dot: CT + TT, blue dot: CC. (b) BMI was plotted against SSB intake in tertiles (three categories) to show the trend of interaction between PEMT-rs72828480 and SSB intake. Orange bar: CT + TT; blue bar: CC.

**Figure 3:**
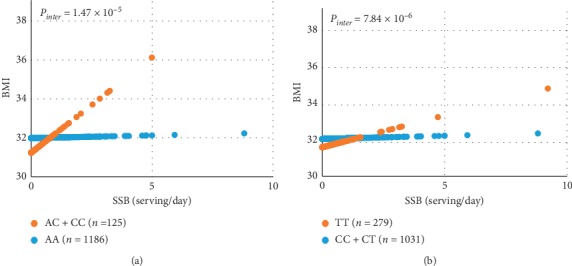
Interaction between *CHKB*-rs75187587 and *ETNK2*-rs1106778 with SSB intake on BMI. *P* values and predicted BMI were calculated while adjusted for sex, age, smoking, alcohol use, physical activity, population structure, and total energy. (a) Predicted BMI was plotted against SSB intake (serving/day) according to *CHKB*-rs75187587 genotype. Orange dot: AC + CC, blue dot: AA. (b) Predicted BMI was plotted against SSB intake (serving/day) according to *ETNK2*-rs1106778 genotype. Orange dot: TT, blue dot: CC + CT.

Considering the important role of PC/PE biosynthetic pathways in metabolic disease [18], we then tested the hypothesis that SSB intake dysregulates these pathways, leading to an increased risk of obesity. Hence, we examined the interaction between SSB intake and genetic variation within the PC/PE biosynthetic pathway on BMI. We identified 83 SNPs in 10 genes involved in this pathway [18]. Of those, 15 SNPs in eight genes exhibited significant interaction with SSB intake influencing BMI (Figure 1 and Table 4), after correction for multiple testing. In particular, three SNPs on the *phosphatidylethanolamine N-methyltransferase* (PEMT) gene showed strong interaction with SSB intake on BMI. Carriers of the rs72828480 T-allele had increased BMI with increased SSB intake (*P*_interaction_=2.06*E* − 07) (Figure 2). Conversely, in CC homozygotes, BMI did not change with higher SSB intake. *Choline kinase beta* (*CHKB*), rs75187587 C–allele carriers showed a strong positive correlation between BMI and SSB intake (Figure 3), whereas AA carriers did not show any change in BMI with increased SSB consumption. Similarly, TT carriers of *ETNK2*-rs1106778 showed an increased risk of obesity when consuming a large amount of SSB, whereas C-allele carriers (CC + CT) did not (Figure 3). Similar interactions were found for other 12 SNPs (data not shown). Overall, the observation that 15 SNPs from eight genes displayed strong interaction with SSB intake on BMI supports the hypothesis that SSB consumption is linked to obesity through PC/PE biosynthetic pathways.

## 4. Discussion

SSB consumption is emerging as a significant factor in the current epidemic of obesity and related metabolic diseases [4, 9, 27]. However, the underlying molecular and metabolic mechanisms that translate consumption into increased adiposity have not been well characterized. In this study, we confirmed that SSB intake was positively associated with obesity in a Caribbean Hispanic population. We then used the plasma metabolite profile as the mediator to link SSB intake to obesity. Based on enrichment analysis of the identified metabolic signatures, two metabolic pathways, PC and LPL, key to the PC/PE biosynthetic pathway, were identified. Therefore, our findings suggest that the PC/PE biosynthetic pathway provides an important link between SSB intake and obesity. Identification of gene-diet interactions with SSB intake focusing on genes in the PC/PE biosynthetic pathway lends further support to the metabolic links between SSB intake and obesity.

Dysregulation in phospholipid metabolism has been linked to obesity and related metabolic diseases [18]. Impairment of LPL metabolism is correlated with obesity and desensitization to *n* − 3 polyunsaturated fatty acids [20]. Alteration of the PC : PE ratio in various tissues can affect energy metabolism and influence the pathology of metabolic disease [18]. Our findings identified metabolic links between SSB intake and obesity, pointing to the PC/PE biosynthetic pathway. SSB intake was positively associated with PC metabolites, which could disrupt the PC : PE molar ratio in the liver, and the hemostasis of phospholipid metabolism, leading to increased risk of obesity.

On the other hand, we also observed a negative association between obesity and PC metabolites, which is consistent with previous reports in other populations [21, 28], suggesting that such correlation could be related to obesity. While obese women have been shown to have lower LPL and PC than women with normal weight, obese women with metabolic syndrome have been shown to have higher lysophospholipids (PC) than healthy obese participants [21]. Hence, SSB intake can dysregulate phospholipid metabolism through the PC/PE biosynthetic pathway, leading to increased risk of other obesity-related diseases, such as nonalcoholic fatty liver disease, type 2 diabetes, and cardiovascular disease.

The PC/PE biosynthetic pathway (see Figure 1) is comprised of two key subpathways: the PEMT and choline pathways [18]. PEMT converts PE to PC, accounting for 30% of PC production from the PEMT pathway, while choline to PC conversion accounts for 70% of PC synthesis in the choline pathway [29]. To illustrate that SSB intake regulates the PC/PE biosynthetic pathway and its association with obesity, we examined whether genetic variation in 10 genes from the PC/PE biosynthetic pathway showed interaction with SSB intake. In the PEMT pathway, PC is converted to PE by the PEMT, where PE can be derived from ethanolamine by ethanolamine kinase (ETNK1 and 2), CTP:phosphoethanolamine cytidylyltransferase (PCYT1A and B), or phosphatidylserine (from mitochondria) by the phosphatidylserine (PS) decarboxylase (PISD).

In particular, we observed strong interaction between *PEMT*-rs72828480 and SSB, and between *ETNK2*–rs1106778 and SSB intake on BMI (Table 4 and Figure 2). PEMT deficiency in Pemt −/− mice showed resistance to high-fat- and high-glucose-induced obesity and diabetes, whereas insufficient PEMT was associated with increased risk of nonalcoholic fatty liver disease [30]. In human studies, a locus near *PEMT* was associated with abdominal obesity and insulin resistance [31]. Decreased *PEMT* expression in omental adipose tissue was correlated with lipid deposition and insulin resistance in obese women [32]. In human thyroid, based on the GTEX database (gtexportal.org), T-allele carriers of *PEMT*-rs72828480 have a significantly lower expression (beta = −0.26, *P*=6.8*E* − 07, *n* = 574) of *PEMT* when compared to C-allele carriers. This observation suggests that T-allele carriers are likely PEMT deficient. In adipose tissue, *PEMT* expression was positively associated with fat mass and BMI [33], and *PEMT*-rs4646343 was highly correlated with *PEMT* expression in adipose tissue [33].

In the choline pathway (Figure 1), choline is converted to PC through CDP-choline by choline kinase (CHKA and B), CYT1A, PCYT1B, and choline/ethanolamine phosphotransferase (CEPT). In this study, we observed that five SNPs in three genes (*CHKB*, *CEPT*, *PCYT1A*) of the choline pathway (Figure 1) exhibited significant interaction with SSB intake on BMI (Table 4). In mice, *CHKB* mutants show reduced phosphocholine and loss of bone mass [34]. In humans, mutations in *CHKB* cause phosphatidylcholine deficiency in myofibers and muscular dystrophy [35]. High-fat feeding and obesity induce *CEPT* expression, whereas *CEPT*-deficient mice have improved insulin sensitivity [36]. In human adipose tissue, *PCYT1A* expression was positively correlated with percent fat mass and BMI [33]. These observations strongly suggest that SSB intake contributes to obesity through PC/PE biosynthetic pathways.

Although it is well established that SSB intake is a cause of obesity [4, 9, 11], we observed a weak association between SSB intake and obesity. However, our observation of a strong GxE interaction of SSB intake on BMI can explain this apparent conundrum. First, we found that 15 SNPs in eight genes of the phospholipid pathway displayed significant GxE interactions with SSB intake on BMI. This implies that the SSB association with obesity is highly dependent on genotype (Figure 2(a), T-allele carriers of *PEMT*-rs72828480 vs CC; Figure 3(a), C-allele carriers of *CHKB*-rs75187587 vs AA; and Figure 3(b), TT of *ETNK2*-rs1106778 vs C-allele carriers) and not solely on SSB intake. Similar findings are known for genotype/nutrient associations with cardiovascular and metabolic diseases depending on nutrients/genotype due to GxE interactions [37, 38]. Second, GxE interaction of SSB intake on obesity depends on the risk allele frequencies. In comparison with other populations, frequencies of risk alleles differ greatly in this population. For instance (Table 4), the risk T-allele of *PEMT*-rs72828480 is more prevalent in this and many European populations, but lower or not present in Asian populations (minor allele frequency (MAF) < 0.001)). Conversely, the risk C-allele of *CHKB*-rs75187587 is rare among Europeans (MAF < 0.005), but it is common in Africans (0.218) and Asians (0.127). We detected significant GxE interaction between *CHKB*-rs75187587 (MAF = 0.05) and SSB intake on obesity. For rs1106778, as this population contains admixed ancestries of Europeans and African and Native Americans [39], the frequency observed in our study (MAF = 0.462) reflects the admixture of Europeans (MAF = 0.337) and Africans (MAF = 0.514). Again, this SNP could have greater impact on obesity in Africans. Overall, although consistent with previous studies reporting that SSB intake is a cause of obesity, our results suggest that SSB intake has a strong impact on obesity through GxE interactions and is not limited to a direct influence on obesity.

Our study is the first to leverage metabolomic data to characterize mechanisms linking SSB intake to obesity through metabolic pathways. Strengths of this study include the following: [1] a comprehensive metabolic profile of a large sample size of middle-aged and older Hispanic participants and [2] integration of metabolome and dietary intake in relation to the outcome and its interaction with genotypes on the outcome. This illustrates how the integration of the genome, metabolome, and environmental factors (nutrients) can help to unravel the underlying mechanisms of how SSB intake may be linked to the pathology of obesity. The current study also has some limitations. First, the metabolome profile was measured in plasma samples. Such metabolomic profiles could differ from those of other tissues, such as the liver and adipose tissues. Second, this cross-sectional study prevents inference of causality between SSB, metabolites, and obesity, although SSB intake is a well-established obesity risk factor [4, 9, 11]. Finally, while our study has identified participants who showed resistance to or predisposition for SSB-induced obesity, our findings are based on one Hispanic population with a high prevalence of obesity; thus, confirmation is required before our findings can be generalized to other populations.

## 5. Conclusions

In conclusion, this study identified two metabolic pathways within the PC/PE biosynthetic pathway, linking SSB intake to obesity. The strong interaction between genetic variants in the identified pathways and SSB consumption on obesity in this study are supported by animal and human studies. Our findings unravel a potential mechanism by which SSB intake increases the risk of obesity.

## Figures and Tables

**Table 1:** General characteristics of all participants and participants with metabolome profile in BPRHS.

	Participants with metabolome	All participants
Participants (*N*)	781	1311
Female, *n* (%)	554 (70.9)	931 (71.0)
Age, mean (SE)	57.3 (0.3)	57.2 (0.2)
BMI, mean (SE)	32.1 (0.2)	31.9 (0.2)
Physical activity score, mean (SE)	31.4 (0.2)	31.4 (0.1)
Smoking		
Current smoking, *n* (%)	180 (23.0)	590 (24.3)
Past smoking, *n* (%)	238 (30.5)	400 (30.5)
Never smoking, *n* (%)	362 (46.3)	319 (45.0)
Alcohol drinking		
Current drinking, *n* (%)	320 (41.0)	381 (39.9)
Past drinking, *n* (%)	236 (30.2)	401 (30.6)
Never drinking, *n* (%)	222 (28.4)	523 (29.1)
Type 2 diabetes, *n* (%)	307 (39.3%)	519 (39.6)
Hypertension, *n* (%)	547 (70.0%)	900 (69.4)
SSB intake, servings/day (SE)	0.49 (0.03)	0.49 (0.02)
Total energy intake (kcal), mean (SE)	2113 (28)	2120 (25)

**Table 2:** Twenty-eight metabolites associated with both SSB intake and BMI.

Metabolites	log (met)^#^ and SSB intake			BMI and SSB intake		
	Beta^*∗*^	SE	*P* value^*∗*^	Beta^*∗*^	SE	*P* value^*∗*^
1-Oleoyl-GPC (18 : 1)	−4.917	0.792	8.80*E* − 10	0.028	0.013	0.030
5-Hydroxylysine	2.001	0.368	7.01*E* − 08	0.047	0.023	0.041
1-Palmitoyl-GPC (16 : 0)	−7.337	1.473	7.84*E* − 07	0.020	0.009	0.030
Glycine	−4.062	0.825	1.04*E* − 06	0.025	0.013	0.047
1,2-Dilinoleoyl-GPC (18 : 2/18 : 2)	−2.976	0.666	9.14*E* − 06	0.039	0.016	0.014
3-Phenylpropionate (hydrocinnamate)	−0.699	0.157	9.97*E* − 06	−0.143	0.061	0.018
1-Linoleoyl-2-linolenoyl-GPC (18 : 2/18 : 3)	−0.910	0.240	1.57*E* − 04	0.076	0.030	0.011
9-Hydroxystearate	1.332	0.354	1.84*E* − 04	0.077	0.027	5.15*E* − 03
Gamma-tocopherol/beta-tocopherol	1.566	0.418	1.95*E* − 04	0.044	0.022	0.044
1-Linoleoyl-GPE (18 : 2)	−1.356	0.368	2.44*E* − 04	0.061	0.019	0.001
1-Oleoyl-GPI (18 : 1)	−1.205	0.339	3.93*E* − 04	0.053	0.025	0.033
1-Oleoyl-GPE (18 : 1)	−1.216	0.366	9.42*E* − 04	0.049	0.021	0.020
1-Palmitoyl-2-stearoyl-GPC (16 : 0/18 : 0)	−2.571	0.783	1.08*E* − 03	0.036	0.012	2.28*E* − 03
2-Hydroxybutyrate/2-hydroxyisobutyrate	1.222	0.376	1.21*E* − 03	−0.068	0.024	4.43*E* − 03
Alpha-ketoglutarate	6.231	1.922	1.24*E* − 03	0.066	0.029	0.021
N-Acetylhistidine	−1.176	0.385	2.32*E* − 03	0.084	0.027	1.93*E* − 03
Butyrylcarnitine (C4)	0.369	0.134	5.83*E* − 03	0.051	0.023	0.025
Cholesterol	−1.103	0.400	5.97*E* − 03	0.030	0.014	0.039
3-Hydroxybutyrylcarnitine (1)	0.629	0.234	7.27*E* − 03	−0.138	0.049	4.92*E* − 03
Methyl-4-hydroxybenzoate sulfate	−0.189	0.071	7.84*E* − 03	0.174	0.080	0.030
1-Stearoyl-2-linoleoyl-GPC (18 : 0/18 : 2)	−2.975	1.218	0.015	0.016	0.008	0.033
1-Palmitoyl-GPC (16 : 1)	−0.893	0.367	0.015	0.071	0.017	4.27*E* − 05
Sphingomyelin (d18 : 2/14 : 0, d18 : 1/14 : 1)	0.706	0.299	0.018	0.048	0.018	0.009
3-Hydroxyisobutyrate	0.882	0.389	0.024	−0.044	0.021	0.036
3-Hydroxybutyrylcarnitine (2)	0.783	0.348	0.025	−0.063	0.026	0.014
Sphingomyelin (d18 : 1/20 : 0, d16 : 1/22 : 0)	−1.620	0.772	0.036	−0.028	0.011	0.011
1,5-Anhydroglucitol (1,5-AG)	−1.086	0.521	0.037	0.090	0.040	0.024
Erythronate	1.684	0.838	0.045	0.026	0.012	0.033

^*∗*^All beta and *P* values were adjusted for age, sex, alcohol use, smoking, education, physical activity, and total energy intake. ^#^Metabolite concentrations were natural log-transformed.

**Table 3:** The ten metabolites that are significantly associated with both SSB intake and BMI and overrepresented in the PC/PE biosynthetic pathways.

Metabolic pathways/metabolite	SSB intake			BMI		
	Beta^*∗*^	SE^*∗*^	*P* value^*∗*^	Beta^*∗*^	SE^*∗*^	*P* value^*∗*^
*Phosphatidylcholine (PC)*						
1-Stearoyl-2-linoleoyl-GPC (18 : 0/18 : 2)	0.016	0.008	0.033	−2.975	1.218	1.48*E* − 02
1,2-Dilinoleoyl-GPC (18 : 2/18 : 2)	0.039	0.016	0.014	−2.976	0.666	9.14*E* − 06
1-Palmitoyl-2-stearoyl-GPC (16 : 0/18 : 0)	0.036	0.012	0.002	−2.571	0.783	1.08*E* − 03
1-Linoleoyl-2-linolenoyl-GPC (18 : 2/18 : 3)	0.076	0.030	0.011	−0.910	0.240	1.57*E* − 04

*Lysophospholipid (LPL)*						
1-Palmitoyl-GPC (16 : 1)	0.071	0.017	4.27*E* − 05	−0.893	0.367	1.53*E* − 02
1-Palmitoyl-GPC (16 : 0)	0.020	0.009	3.03*E* − 02	−7.337	1.473	7.84*E* − 07
1-Oleoyl-GPE (18 : 1)	0.049	0.021	2.02*E* − 02	−1.216	0.366	9.42*E* − 04
1-Linoleoyl-GPE (18 : 2)	0.061	0.019	1.21*E* − 03	−1.356	0.368	2.44*E* − 04
1-Oleoyl-GPI (18 : 1)	0.053	0.025	3.28*E* − 02	−1.205	0.339	3.93*E* − 04
1-Oleoyl-GPC (18 : 1)	0.028	0.013	2.99*E* − 02	−4.917	0.792	8.80*E* − 10

^*∗*^All *P*, beta, and SE values were estimated based on a linear regression model adjusted for age, sex, smoking, alcohol use, and physical activity score.

**Table 4:** Interaction between SSB intake and SNPs in 10 genes of PC/PE biosynthetic pathways on BMI.

Gene	SNP	Chr	Position	*P*-interaction^*∗*^	FDR	Beta (GxE)^#^	SE (Beta_GxE_)	Minor/Major	MAF
*CEPT1*	rs12745827	1	111700001	1.44*E* − 03	1.71*E* − 02	1.746	0.547	G/T	0.074
*CHKB*	rs75187587	22	51019113	**1.47E − 05**	6.1**0E − 04**	2.522	0.580	C/A	0.050
*CHKB*	rs86337	22	51020668	2.03*E* − 03	2.11*E* − 02	−1.017	0.329	C/A	0.375
*CHKB*	rs6009931	22	51023152	7.50*E* − 03	4.44*E* − 02	1.444	0.539	G/T	0.076
*ETNK1*	rs17427520	12	22833518	2.76*E* − 04	5.73*E* − 03	2.048	0.561	C/T	0.021
*ETNK1*	rs2271097	12	22837282	2.87*E* − 03	2.97*E* − 02	1.797	0.602	G/A	0.040
*ETNK2*	rs1106778	1	204115548	**7.84E − 06**	**3.25E − 04**	1.399	0.312	T/C	0.462
*ETNK2*	rs4951313	1	204122978	1.07*E* − 03	1.48*E* − 02	−1.012	0.309	G/A	0.371
*PCYT1A*	rs61588443	3	196013318	4.82*E* − 03	3.33*E* − 02	0.889	0.315	G/A	0.262
*PEMT*	rs72828480	17	17442854	**3.34E − 07**	**2.77E − 05**	3.548	0.692	T/C	0.031
*PEMT*	rs2124344	17	17480195	1.17*E* − 03	1.39*E* − 02	1.262	0.388	A/G	0.363
*PEMT*	rs66637059	17	17417808	9.34*E* − 04	1.55*E* − 02	1.557	0.469	T/C	0.118
*PISD*	rs11703808	22	32024980	4.21*E* − 03	3.18*E* − 02	0.953	0.332	G/A	0.479

^*∗*^ *P*-interaction: *P* values of interaction between SNP and SSB intake on BMI. ^#^Beta_(GxE)_: beta of interaction between SNP and SSB model adjusted for age, sex, smoking, alcohol use, physical activity score, and population structure. MAF: minor allele frequency; FDR: false discovery rate; sample size: *n* = 1311.

## Data Availability

The data used to support the findings of this study are available from the corresponding author upon request.

## Supplementary Materials

Supplementary Materials Supplementary Figure 1: flowchart of BPRHS sample for metabolomics analysis. Table S1: association analysis of metabolites with BMI and/or SSB intake. Table S2: metabolic pathways enrichment by SSB intake. Table S3: metabolic pathways overrepresented by BMI. Table S4: pathway enrichment analysis for common metabolites that are associated with both SSB intake and BMI.
