# Supplementary material for: Metabolomic Links between Sugar-Sweetened Beverage Intake and Obesity
Source: J Obes. 2020 Apr 13;2020:7154738. doi: 10.1155/2020/7154738 (PMC7211252; doi:10.1155/2020/7154738)
Supplement: Supplementary Materials — Supplementary Figure 1: flowchart of BPRHS sample for metabolomics analysis. Table S1: association analysis of metabolites with BMI and/or SSB intake. Table S2: metabolic pathways enrichment by SSB intake. Table S3: metabolic pathways overrepresented by BMI. Table S4: pathway enrichment analysis for common metabolites that are associated with both SSB intake and BMI. [file 7154738.f1.pdf]

Metabolomic links between sugar-sweetened beverage intake and obesity

Bingjie Zhou, Reiko Ichikawa, Laurence D. Parnell, Sabrina E. Noel, Xiyuan Zhang, Shilpa N. Bhupathiraju, Caren E. Smith, Katherine L. Tucker, Jose M. Ordovas, Chao-Qiang Lai

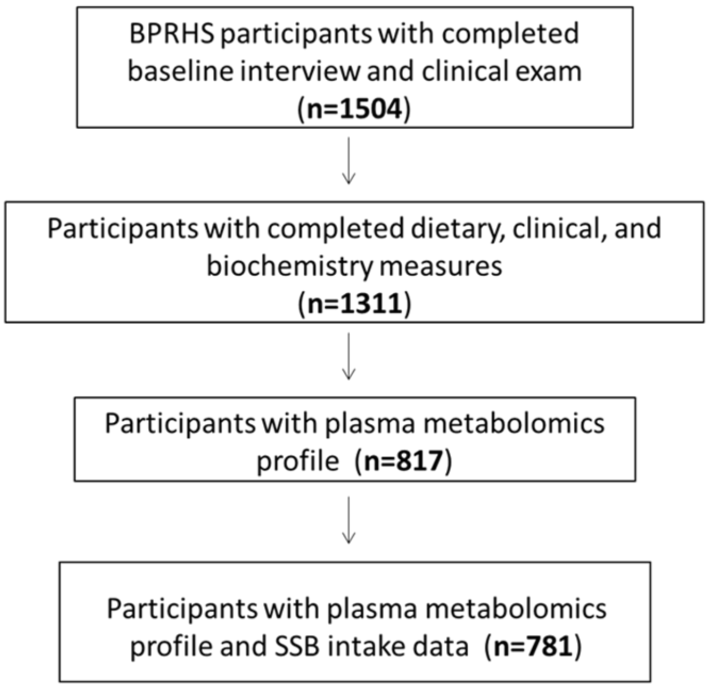

Supplemental Figure 1. Flowchart of BPRHS sample for metabolomics analysis

Table S1. Association analysis of metabolites with BMI and/or SSB intake

[Type here]		BMI			SSB		
CompID	BIOCHEMICAL	Beta*	SE	P value*	Beta*	SE	P value*
54805	3beta-hydroxy-5-cholestenoate	-5.559	0.666	3.15E-16	-0.003	0.018	0.86
48153	mannose	3.756	0.466	2.93E-15	-0.014	0.015	0.321
15506	choline	7.828	1.141	1.40E-11	0.008	0.009	0.373
32418	myristoleate (14:1n5)	2.344	0.352	5.07E-11	0.045	0.026	0.081
33447	palmitoleate (16:1n7)	2.011	0.31	1.53E-10	0.033	0.03	0.274
1604	urate	4.962	0.794	6.85E-10	0.003	0.01	0.741
48258	1-oleoyl-GPC (18:1)	-4.917	0.792	8.80E-10	0.028	0.013	0.03
43829	gamma-glutamylvaline	5.626	0.935	2.80E-09	0.005	0.016	0.732
44526	3-methyl-2-oxobutyrate	3.103	0.527	5.89E-09	-0.017	0.011	0.141
33422	gamma-glutamylphenylalanine	5.604	0.96	7.92E-09	0.012	0.013	0.354
33971	10-heptadecenoate (17:1n7)	2.355	0.426	4.41E-08	0.026	0.024	0.278
34419	1-linoleoyl-GPC (18:2)	-4.159	0.754	4.85E-08	0.019	0.012	0.109
15685	5-hydroxylysine	2.001	0.368	7.01E-08	0.047	0.023	0.041
59	histidine	-8.330	1.555	1.13E-07	0.007	0.007	0.306
512	asparagine	-4.151	0.791	2.00E-07	0.000	0.007	0.986
57	glutamate	8.624	1.669	3.02E-07	0.022	0.018	0.228
1417	kynurenate	2.048	0.397	3.15E-07	-0.008	0.02	0.707
1299	tyrosine	3.907	0.758	3.29E-07	0.014	0.011	0.174
33961	1-stearoyl-GPC (18:0)	-6.249	1.236	5.35E-07	0.013	0.011	0.233
33955	1-palmitoyl-GPC (16:0)	-7.337	1.473	7.84E-07	0.020	0.009	0.03
58	glycine	-4.062	0.825	1.04E-06	0.025	0.013	0.047
34407	isovalerylcarnitine (C5)	1.936	0.412	3.14E-06	-0.020	0.019	0.274
52285	oleate/vaccenate (18:1)	2.532	0.54	3.21E-06	-0.005	0.023	0.825
64	phenylalanine	7.470	1.595	3.33E-06	0.001	0.006	0.9

35136	5-methyluridine (ribothymidine)	-4.663	1.038	8.07E-06	-0.008	0.009	0.407
52603	1,2-dilinoleoyl-GPC (18:2/18:2)	-2.976	0.666	9.14E-06	0.039	0.016	0.014
57330	lignoceroyl sphingomyelin (d18:1/24:0)	-2.243	0.504	9.65E-06	-0.003	0.018	0.85
15749	3-phenylpropionate (hydrocinnamate)	-0.699	0.157	9.97E-06	-0.143	0.061	0.018
1365	myristate (14:0)	2.483	0.562	1.15E-05	0.014	0.019	0.466
1561	alpha-tocopherol	-2.130	0.495	1.93E-05	0.016	0.026	0.542
34035	linolenate [alpha or gamma; (18:3n3 or 6)]	2.185	0.513	2.35E-05	-0.015	0.028	0.576
33972	10-nonadecenoate (19:1n9)	1.904	0.454	3.11E-05	0.012	0.024	0.605
1336	palmitate (16:0)	3.527	0.843	3.20E-05	-0.002	0.016	0.893
1105	linoleate (18:2n6)	2.597	0.653	7.71E-05	-0.006	0.023	0.788
40499	4-hydroxyglutamate	0.979	0.25	0.000101	0.060	0.043	0.165
18369	gamma-glutamylleucine	3.127	0.802	0.000104	-0.006	0.014	0.638
40473	hydantoin-5-propionic acid	0.739	0.191	0.000117	0.001	0.035	0.973
34409	stearoylcarnitine (C18)	-2.133	0.551	0.000118	0.020	0.017	0.248
42459	sphingomyelin (d18:2/16:0, d18:1/16:1)	3.326	0.865	0.000131	0.016	0.01	0.111
32980	adrenate (22:4n6)	1.516	0.399	0.000155	-0.015	0.025	0.546
53176	1-linoleoyl-2-linolenoyl-GPC (18:2/18:3)	-0.910	0.24	0.000157	0.076	0.03	0.011
47120	9-hydroxystearate	1.332	0.354	0.000184	0.077	0.027	0.00515
52473	gamma-tocopherol/beta-tocopherol	1.566	0.418	0.000195	0.044	0.022	0.044
1301	lysine	4.186	1.131	0.00023	0.003	0.008	0.719
36600	1-linoleoyl-GPE (18:2)	-1.356	0.368	0.000244	0.061	0.019	0.00121
38661	hydroxycotinine	-0.773	0.21	0.000253	-0.042	0.042	0.325
52478	1-(1-enyl-palmitoyl)-2-oleoyl-GPC (P-16:0/18:1)	-3.578	0.979	0.000274	0.007	0.014	0.619

37506	palmitoyl sphingomyelin (d18:1/16:0)	-5.266	1.443	0.00028	0.001	0.007	0.92
17805	dihomo-linoleate (20:2n6)	1.817	0.504	0.00033	-0.009	0.023	0.705
40008	isoleucylglycine	-7.685	2.132	0.000333	0.053	0.029	0.067
1589	N-acetylmethionine	2.010	0.559	0.000346	0.004	0.015	0.776
37058	succinylcarnitine (C4-DC)	1.996	0.556	0.000355	0.027	0.016	0.094
52710	1-linoleoyl-2-arachidonoyl-GPC (18:2/20:4n6)	-1.913	0.536	0.000381	0.006	0.015	0.685
48351	N1-methylinosine	1.906	0.535	0.000393	0.027	0.016	0.103
36602	1-oleoyl-GPI (18:1)	-1.205	0.339	0.000393	0.053	0.025	0.033
36103	p-cresol sulfate	-0.666	0.192	0.00054	-0.012	0.046	0.801
15720	N-acetylglutamate	1.906	0.569	0.000856	0.022	0.033	0.498
55015	gamma-glutamyl-alpha-lysine	1.898	0.57	0.000921	0.008	0.013	0.54
35628	1-oleoyl-GPE (18:1)	-1.216	0.366	0.000942	0.049	0.021	0.02
52616	1-palmitoyl-2-stearoyl-GPC (16:0/18:0)	-2.571	0.783	0.00108	0.036	0.012	0.00228
52281	2-hydroxybutyrate/2-hydroxyisobutyrate	1.222	0.376	0.00121	-0.068	0.024	0.00443
528	alpha-ketoglutarate	6.231	1.922	0.00124	0.066	0.029	0.021
53242	5-bromotryptophan	-1.580	0.493	0.00142	0.029	0.018	0.105
33968	5-dodecenoate (12:1n7)	1.167	0.367	0.00154	0.021	0.025	0.408
443	aspartate	10.676	3.372	0.00161	-0.002	0.014	0.856
38754	gamma-carboxyglutamate	1.898	0.61	0.00193	0.005	0.015	0.737
32506	2-linoleoylglycerol (18:2)	-0.550	0.179	0.00215	0.024	0.036	0.502
27665	1-methylnicotinamide	-1.219	0.396	0.00219	-0.040	0.022	0.073
33946	N-acetylhistidine	-1.176	0.385	0.00232	0.084	0.027	0.00193
35157	N6-carbamoylthreonyladenosine	1.519	0.498	0.00235	0.024	0.015	0.124
27731	ribonate	1.228	0.412	0.00294	0.027	0.02	0.164
33969	stearidonate (18:4n3)	1.708	0.575	0.00309	-0.011	0.029	0.704
42398	1-stearoyl-GPE (18:0)	-1.876	0.633	0.00314	0.020	0.017	0.249

1898	proline	3.019	1.045	0.00396	0.002	0.009	0.866
52988	adipoylcarnitine (C6-DC)	0.655	0.228	0.00411	0.000	0.025	0.986
43582	5-(galactosylhydroxy)-L-lysine	0.934	0.326	0.00432	0.087	0.054	0.104
37529	sphingomyelin (d18:1/18:1, d18:2/18:0)	2.134	0.747	0.00437	-0.017	0.011	0.131
41220	2-stearoyl-GPE (18:0)	-1.785	0.626	0.0045	0.025	0.021	0.234
52474	1-(1-enyl-palmitoyl)-GPC (P-16:0)	-3.079	1.088	0.00476	0.022	0.02	0.273
36098	4-vinylphenol sulfate	-0.689	0.243	0.00478	-0.017	0.047	0.723
37752	13-HODE + 9-HODE	106.517	37.656	0.0048	0.050	0.027	0.067
52435	sphingomyelin (d18:2/23:0, d18:1/23:1, d17:1/24:1)	-1.008	0.359	0.00515	-0.007	0.02	0.724
33587	eicosenoate (20:1)	1.341	0.479	0.00529	-0.019	0.025	0.446
553	cotinine	-1.189	0.425	0.00531	-0.011	0.074	0.887
1118	arachidate (20:0)	-3.755	1.346	0.00539	0.015	0.01	0.133
54923	beta-citrylglutamate	1.253	0.452	0.00569	-0.007	0.016	0.654
32412	butyrylcarnitine (C4)	0.369	0.134	0.00583	0.051	0.023	0.025
63	cholesterol	-1.103	0.4	0.00597	0.030	0.014	0.039
47153	sphingomyelin (d18:1/24:1, d18:2/24:0)	-1.605	0.585	0.0062	0.024	0.013	0.079
1868	cysteine	0.561	0.205	0.0063	0.011	0.013	0.382
48492	behenoyl sphingomyelin (d18:1/22:0)	-1.840	0.681	0.00703	-0.003	0.013	0.81
43264	3-hydroxybutyrylcarnitine (1)	0.629	0.234	0.00727	-0.138	0.049	0.00492
42382	S-adenosylhomocysteine (SAH)	2.527	0.942	0.00747	0.035	0.022	0.119
48429	methyl-4-hydroxybenzoate sulfate	-0.189	0.071	0.00784	0.174	0.08	0.03
19263	1-palmitoyl-2-oleoyl-GPE (16:0/18:1)	0.474	0.178	0.00805	0.032	0.028	0.243
36738	gamma-glutamylglutamate	1.986	0.748	0.00808	0.018	0.022	0.411
52436	tricosanoyl sphingomyelin (d18:1/23:0)	-0.998	0.384	0.01	-0.018	0.017	0.297

36845	o-cresol sulfate	-0.716	0.277	0.01	-0.026	0.055	0.639
60	leucine	2.577	1.037	0.013	-0.008	0.008	0.361
1591	N-acetylvaline	1.974	0.794	0.013	0.008	0.011	0.444
1638	arginine	-3.401	1.373	0.013	0.009	0.01	0.356
2132	citrulline	-2.138	0.864	0.014	-0.001	0.011	0.949
37097	tryptophan betaine	-0.484	0.197	0.014	-0.064	0.063	0.305
39600	3-hydroxyhippurate	0.108	0.044	0.015	-0.001	0.063	0.984
52452	1-stearoyl-2-linoleoyl-GPC (18:0/18:2)	-2.975	1.218	0.015	0.016	0.008	0.033
43231	6-oxopiperidine-2-carboxylate	1.061	0.436	0.015	0.013	0.021	0.522
18477	glycodeoxycholate	-0.547	0.225	0.015	0.046	0.116	0.689
33230	1-palmitoleoyl-GPC (16:1)	-0.893	0.367	0.015	0.071	0.017	4.27E-05
35631	1-palmitoyl-GPE (16:0)	-1.265	0.526	0.016	0.028	0.018	0.115
52975	glycodeoxycholate sulfate	-0.627	0.265	0.018	0.020	0.056	0.727
47154	sphingomyelin (d18:2/14:0, d18:1/14:1)	0.706	0.299	0.018	0.048	0.018	0.00881
52673	1-(1-enyl-palmitoyl)-2-arachidonoyl-GPE (P-16:0/20:4)	1.112	0.47	0.018	-0.014	0.019	0.455
37431	N-methylproline	0.331	0.141	0.019	0.057	0.065	0.386
52464	1-palmitoyl-2-arachidonoyl-GPE (16:0/20:4)	0.684	0.293	0.02	0.002	0.022	0.911
6146	2-aminoadipate	0.762	0.327	0.02	0.002	0.033	0.951
32197	3-(4-hydroxyphenyl)lactate	1.241	0.534	0.021	0.022	0.015	0.15
34389	1-methylxanthine	-0.245	0.106	0.021	0.041	0.053	0.44
1549	3-hydroxyisobutyrate	0.882	0.389	0.024	-0.044	0.021	0.036
35186	1-arachidonoyl-GPE (20:4n6)	-1.263	0.557	0.024	0.015	0.014	0.278
52984	3-hydroxybutyrylcarnitine (2)	0.783	0.348	0.025	-0.063	0.026	0.014
1494	5-oxoproline	-6.076	2.701	0.025	0.001	0.01	0.94
37482	17alpha-hydroxypregnenolone 3-sulfate	-0.660	0.295	0.026	-0.021	0.043	0.631

15677	3-methylhistidine	0.293	0.131	0.026	-0.049	0.052	0.344
1648	serine	-2.852	1.283	0.027	-0.001	0.01	0.909
15443	glucuronate	0.952	0.428	0.027	0.042	0.032	0.193
17945	2-hydroxystearate	-2.398	1.087	0.028	0.000	0.011	0.97
42574	glycohyocholate	-0.273	0.126	0.03	0.053	0.054	0.334
33228	1-arachidonoyl-GPC (20:4n6)	-1.086	0.503	0.031	-0.006	0.017	0.716
39271	1-(1-enyl-stearoyl)-GPE (P-18:0)	-1.751	0.815	0.032	-0.005	0.023	0.824
45095	2-methylbutyrylcarnitine (C5)	0.846	0.394	0.032	-0.021	0.03	0.495
52495	sphingomyelin (d18:1/21:0, d17:1/22:0, d16:1/23:0)	-0.655	0.306	0.033	-0.019	0.018	0.281
53	glutamine	-2.039	0.952	0.033	0.004	0.007	0.524
396	glutarate (C5-DC)	0.893	0.42	0.034	0.041	0.05	0.42
32388	dodecanedioate (C12-DC)	-0.647	0.304	0.034	0.036	0.025	0.146
32504	docosapentaenoate (n3 DPA; 22:5n3)	1.024	0.485	0.035	-0.029	0.024	0.228
1493	ornithine	1.777	0.845	0.036	0.014	0.012	0.246
12261	taurodeoxycholate	-0.181	0.086	0.036	0.035	0.078	0.656
48490	sphingomyelin (d18:1/20:0, d16:1/22:0)	-1.620	0.772	0.036	-0.028	0.011	0.011
20675	1,5-anhydroglucitol (1,5-AG)	-1.086	0.521	0.037	0.090	0.04	0.024
1125	isoleucine	2.163	1.043	0.038	-0.008	0.009	0.36
42449	1-palmitoyl-2-linoleoyl-GPE (16:0/18:2)	0.444	0.216	0.04	0.039	0.026	0.135
53223	palmitoleoylcarnitine (C16:1)	0.573	0.279	0.041	0.028	0.022	0.215
1121	margarate (17:0)	1.409	0.695	0.043	0.002	0.017	0.912
55017	4-hydroxyphenylacetylglutamine	0.168	0.083	0.044	0.071	0.047	0.134
42420	erythronate	1.684	0.838	0.045	0.026	0.012	0.033
36847	2-ethylphenylsulfate	-0.671	0.334	0.045	-0.061	0.032	0.055
35675	2-hydroxypalmitate	-2.347	1.186	0.048	0.002	0.01	0.817

52447	1-stearoyl-2-arachidonoyl-GPE (18:0/20:4)	0.674	0.341	0.049	-0.021	0.019	0.276
52726	1-stearoyl-2-oleoyl-GPI (18:0/18:1)	-0.450	0.23	0.051	0.111	0.034	0.000929
52748	1-(1-enyl-stearoyl)-2-linoleoyl-GPE (P-18:0/18:2)	-0.896	0.466	0.055	0.042	0.018	0.018
52468	1-stearoyl-2-linoleoyl-GPI (18:0/18:2)	-0.897	0.469	0.056	0.066	0.018	0.00032
35305	1-palmitoyl-GPI (16:0)	-0.663	0.349	0.058	0.105	0.027	8.02E-05
45951	1-linolenoyl-GPC (18:3)	-0.496	0.271	0.068	0.060	0.026	0.021
36594	1-linoleoyl-GPI (18:2)	-0.695	0.386	0.072	0.040	0.018	0.024
32346	glycochenodeoxycholate	-0.333	0.186	0.074	0.110	0.049	0.024
52461	1-palmitoyl-2-oleoyl-GPC (16:0/18:1)	-1.477	0.836	0.078	0.037	0.01	0.000311
52615	sphingomyelin (d18:1/17:0, d17:1/18:0, d19:1/16:0)	-1.175	0.668	0.079	-0.034	0.012	0.0057
42374	2-aminobutyrate	0.985	0.573	0.086	-0.060	0.016	0.00014
53260	oleoylcholine	-0.043	0.025	0.089	0.155	0.056	0.00585
32390	N-acetyltyrosine	0.554	0.341	0.105	0.060	0.023	0.00956
52449	1-stearoyl-2-arachidonoyl-GPI (18:0/20:4)	-0.846	0.538	0.116	0.033	0.015	0.024
33950	N-acetylphenylalanine	0.510	0.328	0.12	0.051	0.021	0.013
33364	gamma-glutamylthreonine	1.442	0.935	0.124	0.037	0.016	0.021
52438	1-stearoyl-2-oleoyl-GPC (18:0/18:1)	-0.717	0.479	0.135	0.067	0.015	5.98E-06
31591	androsterone sulfate	-0.674	0.455	0.139	-0.121	0.052	0.02
54967	palmitoleoyl-linoleoyl-glycerol (16:1/18:2) [1]	0.325	0.223	0.145	0.061	0.03	0.043
39379	glycoursodeoxycholate	-0.218	0.151	0.149	0.166	0.058	0.00445
52944	palmitoylcholine	-0.052	0.037	0.162	0.118	0.045	0.0081

18494	taurochenodeoxycholate	-0.060	0.045	0.183	0.137	0.061	0.025
52470	1-palmitoyl-2-palmitoleoyl-GPC (16:0/16:1)	0.282	0.223	0.206	0.098	0.023	2.37E-05
42446	1-palmitoyl-2-linoleoyl-GPC (16:0/18:2)	-1.575	1.261	0.212	0.015	0.008	0.05
18476	glycocholate	-0.128	0.103	0.215	0.137	0.055	0.012
46115	21-hydroxypregnenolone disulfate	-0.418	0.358	0.244	-0.081	0.03	0.00762
19130	1,2-dipalmitoyl-GPC (16:0/16:0)	-1.128	0.969	0.245	0.037	0.01	0.000101
48434	N-acetylcitrulline	-0.017	0.015	0.248	0.118	0.046	0.011
53261	arachidonoylcholine	-0.034	0.029	0.251	0.096	0.047	0.043
48460	propyl 4-hydroxybenzoate sulfate	-0.042	0.037	0.26	0.159	0.077	0.038
33973	epiandrosterone sulfate	-0.707	0.641	0.27	-0.163	0.052	0.00193
40703	prolylglycine	-1.052	1.035	0.31	0.088	0.043	0.04
15990	glycerophosphorylcholine (GPC)	-0.938	0.99	0.344	0.060	0.024	0.013
22185	N-acetylaspartate (NAA)	0.208	0.245	0.397	0.164	0.05	0.00113
35428	tiglylcarnitine (C5:1-DC)	0.381	0.489	0.436	-0.040	0.018	0.029
33959	N-acetyltryptophan	0.271	0.371	0.465	0.059	0.02	0.00359
47112	etiocholanolone glucuronide	-0.269	0.368	0.465	-0.102	0.04	0.011
54942	palmitoyl-oleoyl-glycerol (16:0/18:1)	0.216	0.299	0.469	0.092	0.044	0.038
35127	pro-hydroxy-pro	-0.165	0.236	0.485	0.079	0.024	0.000861
21127	1-palmitoylglycerol (16:0)	-0.215	0.351	0.539	0.058	0.027	0.034
36808	dimethylarginine (SDMA + ADMA)	0.568	0.992	0.567	0.018	0.008	0.025
37211	androstenediol (3beta,17beta) monosulfate (1)	0.289	0.505	0.568	-0.106	0.041	0.00876
33936	octanoylcarnitine (C8)	0.058	0.107	0.592	-0.068	0.029	0.018
1414	3-phosphoglycerate	-0.197	0.37	0.595	-0.108	0.051	0.033
19503	stearoyl sphingomyelin (d18:1/18:0)	0.387	0.755	0.608	-0.037	0.012	0.00226

48491	sphingomyelin (d18:1/20:1, d18:2/20:0)	-0.347	0.699	0.62	-0.028	0.013	0.027
37186	5alpha-androstan-3alpha,17beta-diol monosulfate (1)	0.170	0.355	0.632	-0.140	0.054	0.01
32425	dehydroisoandrosterone sulfate (DHEA-S)	-0.293	0.691	0.672	-0.091	0.034	0.00795
48885	arabitol/xylitol	-0.109	0.266	0.681	0.046	0.017	0.00645
48408	tyramine O-sulfate	-0.053	0.136	0.695	0.103	0.051	0.044
55001	diacylglycerol (12:0/18:1, 14:0/16:1, 16:0/14:1)	0.035	0.091	0.701	0.156	0.071	0.028
1498	N6,N6,N6-trimethyllysine	-0.101	0.313	0.748	0.046	0.022	0.039
37190	5alpha-androstan-3beta,17beta-diol disulfate	0.180	0.624	0.773	-0.097	0.049	0.046
45970	1-palmitoyl-GPG (16:0)	0.114	0.501	0.819	0.058	0.017	0.000912
22842	cholate	0.012	0.074	0.872	0.219	0.067	0.00116
37192	5alpha-androstan-3beta,17beta-diol monosulfate (2)	-0.063	0.555	0.91	-0.129	0.048	0.00725
52431	1-palmitoleoylglycerol (16:1)	0.017	0.169	0.919	0.108	0.038	0.00523
46957	gulonate	0.022	0.255	0.931	0.124	0.048	0.011
1123	inosine	-0.008	0.278	0.977	0.087	0.032	0.00634
52467	1-palmitoyl-2-arachidonoyl-GPI (16:0/20:4)	-0.004	0.222	0.987	0.114	0.026	8.97E-06

*All were adjusted for age, sex, alcohol use, smoking, education, physical activity, and total calorie intake.

Table S2. Metabolic pathways enrichment by SSB intake

Sub Pathway	Super Pathway	No. of metabolites assigined to the pathway	No. of significant metabolites in each pathway	Total No. of significant metabolites	Z score	P value
Phosphatidylcholine (PC)	Lipid	12	8	86	**4.109**	**3.97E-05**
Phosphatidylinositol (PI)	Lipid	4	4	86	**4.026**	**5.68E-05**
Lysophospholipid (LPL)	Lipid	19	10	86	**3.649**	**2.63E-04**
Fatty Acid Metabolism (Acyl Choline)	Lipid	3	3	86	**3.482**	**4.97E-04**
Androgenic Steroids	Lipid	17	8	86	2.857	4.28E-03
Primary Bile Acid Metabolism	Lipid	8	4	86	2.149	3.16E-02
Glutathione Metabolism	Amino Acid	3	2	86	2.033	4.21E-02
Glycolysis, Gluconeogenesis, and Pyruvate Metabolism	Carbohydrate	3	2	86	2.033	4.21E-02
Xanthine Metabolism	Xenobiotics	14	0	86	-1.894	5.82E-02
Long Chain Fatty Acid	Lipid	12	0	86	-1.750	8.02E-02
Polyunsaturated Fatty Acid (n3 and n6)	Lipid	12	0	86	-1.750	8.02E-02
Methionine, Cysteine, SAM and Taurine Metabolism	Amino Acid	11	0	86	-1.673	9.43E-02
Fatty Acid, Dicarboxylate	Lipid	9	0	86	-1.510	1.31E-01
Glutamate Metabolism	Amino Acid	9	0	86	-1.510	1.31E-01
Pyrimidine Metabolism, Uracil containing	Nucleotide	6	0	86	-1.228	2.19E-01
Phosphatidylethanolamine (PE)	Lipid	6	0	86	-1.228	2.19E-01

Leucine, Isoleucine and Valine Metabolism	Amino Acid	20	2	86	-1.135	2.56E-01
Secondary Bile Acid Metabolism	Lipid	12	1	86	-1.017	3.09E-01
Progestin Steroids	Lipid	4	0	86	-1.001	3.17E-01
Fructose, Mannose and Galactose Metabolism	Carbohydrate	4	0	86	-1.001	3.17E-01
Lysoplasmalogen	Lipid	4	0	86	-1.001	3.17E-01
Tryptophan Metabolism	Amino Acid	11	1	86	-0.909	3.63E-01
Nicotinate and Nicotinamide Metabolism	Cofactors and Vitamins	3	0	86	-0.866	3.87E-01
Purine Metabolism, Adenine containing	Nucleotide	3	0	86	-0.866	3.87E-01
Medium Chain Fatty Acid	Lipid	3	0	86	-0.866	3.87E-01
Acetylated Peptides	Peptide	3	0	86	-0.866	3.87E-01
Dihydrosphingomyelins	Lipid	3	0	86	-0.866	3.87E-01
Tobacco Metabolite	Xenobiotics	3	0	86	-0.866	3.87E-01
Sphingomyelins	Lipid	18	5	86	0.853	3.94E-01
Fatty Acid, Monohydroxy	Lipid	10	1	86	-0.793	4.28E-01
Plasmalogen	Lipid	10	1	86	-0.793	4.28E-01
Gamma-glutamyl Amino Acid	Peptide	10	1	86	-0.793	4.28E-01
Histidine Metabolism	Amino Acid	9	1	86	-0.667	5.05E-01
Ascorbate and Aldarate Metabolism	Cofactors and Vitamins	3	1	86	0.584	5.59E-01
Phenylalanine Metabolism	Amino Acid	3	1	86	0.584	5.59E-01
Fatty Acid Metabolism (also BCAA Metabolism)	Lipid	3	1	86	0.584	5.59E-01
Aminosugar Metabolism	Carbohydrate	3	1	86	0.584	5.59E-01

Sterol	Lipid	3	1	86	0.584	5.59E-01
Dipeptide	Peptide	3	1	86	0.584	5.59E-01
Tyrosine Metabolism	Amino Acid	14	2	86	-0.535	5.93E-01
Glycine, Serine and Threonine Metabolism	Amino Acid	8	1	86	-0.529	5.97E-01
Benzoate Metabolism	Xenobiotics	19	3	86	-0.459	6.46E-01
Urea cycle; Arginine and Proline Metabolism	Amino Acid	12	3	86	0.448	6.54E-01
Alanine and Aspartate Metabolism	Amino Acid	4	1	86	0.256	7.98E-01
Pentose Metabolism	Carbohydrate	4	1	86	0.256	7.98E-01
Tocopherol Metabolism	Cofactors and Vitamins	4	1	86	0.256	7.98E-01
Purine Metabolism, (Hypo)Xanthine/Inosine containing	Nucleotide	6	1	86	-0.200	8.42E-01
TCA Cycle	Energy	6	1	86	-0.200	8.42E-01
Monoacylglycerol	Lipid	9	2	86	0.176	8.61E-01
Lysine Metabolism	Amino Acid	9	2	86	0.176	8.61E-01
Fatty Acid Metabolism(Acyl Carnitine)	Lipid	16	3	86	-0.118	9.06E-01
Diacylglycerol	Lipid	15	3	86	0.009	9.93E-01
Pregnenolone Steroids	Lipid	5	1	86	0.005	9.96E-01
Phospholipid Metabolism	Lipid	5	1	86	0.005	9.96E-01

[1]Number of metabolites are the number of detected metabolites assigned to the respective pathway.

Table S3. Metabolic pathways overpresenetd by BMI

**Sub Pathway**	**Super Pathway**	**No. of metabolites assigined to the pathway**	**No. of significant metabolites in each pathway**	**Total No. of significant metabolites**	**Z score**	**P value**
Long Chain Fatty Acid	Lipid	12	10	148	**3.629**	**2.85E-04**
Lysophospholipid	Lipid	19	13	148	3.205	1.35E-03
Sphingomyelins	Lipid	18	11	148	2.449	1.43E-02
Xanthine Metabolism	Xenobiotics	14	1	148	-2.171	2.99E-02
Glutamate Metabolism	Amino Acid	9	6	148	2.068	3.87E-02
Phosphatidylethanolamine (PE)	Lipid	6	4	148	1.682	9.25E-02
Tyrosine Metabolism	Amino Acid	14	2	148	-1.599	1.10E-01
Monoacylglycerol	Lipid	9	1	148	-1.477	1.40E-01
Lysine Metabolism	Amino Acid	9	5	148	1.359	1.74E-01
Aminosugar Metabolism	Carbohydrate	3	2	148	1.185	2.36E-01
Glutathione Metabolism	Amino Acid	3	2	148	1.185	2.36E-01
Sterol	Lipid	3	2	148	1.185	2.36E-01
Tobacco Metabolite	Xenobiotics	3	2	148	1.185	2.36E-01
Polyunsaturated Fatty Acid (n3 and n6)	Lipid	12	6	148	1.164	2.44E-01
Gamma-glutamyl Amino Acid	Peptide	10	5	148	1.060	2.89E-01
Plasmalogen	Lipid	10	2	148	-0.960	3.37E-01
Pyrimidine Metabolism, Uracil containing	Nucleotide	6	1	148	-0.913	3.61E-01

Fatty Acid, Dicarboxylate	Lipid	9	2	148	-0.768	4.42E-01
Phospholipid Metabolism	Lipid	5	1	148	-0.675	5.00E-01
Pregnenolone Steroids	Lipid	5	1	148	-0.675	5.00E-01
Alanine and Aspartate Metabolism	Amino Acid	4	2	148	0.666	5.06E-01
Lysoplasmalogen	Lipid	4	2	148	0.666	5.06E-01
Tocopherol Metabolism	Cofactors and Vitamins	4	2	148	0.666	5.06E-01
Histidine Metabolism	Amino Acid	9	4	148	0.650	5.16E-01
Glycine, Serine and Threonine Metabolism	Amino Acid	8	2	148	-0.556	5.78E-01
Phosphatidylcholine (PC)	Lipid	12	5	148	0.548	5.84E-01
Urea cycle; Arginine and Proline Metabolism	Amino Acid	12	5	148	0.548	5.84E-01
Methionine, Cysteine, SAM and Taurine Metabolism	Amino Acid	11	3	148	-0.494	6.21E-01
Tryptophan Metabolism	Amino Acid	11	3	148	-0.494	6.21E-01
Fructose, Mannose and Galactose Metabolism	Carbohydrate	4	1	148	-0.392	6.95E-01
Pentose Metabolism	Carbohydrate	4	1	148	-0.392	6.95E-01
Fatty Acid, Monohydroxy	Lipid	10	4	148	0.387	6.99E-01
Fatty Acid Metabolism(Acyl Carnitine)	Lipid	16	5	148	-0.258	7.96E-01
Benzoate Metabolism	Xenobiotics	19	7	148	0.242	8.09E-01

Leucine, Isoleucine and Valine Metabolism	Amino Acid	20	7	148	0.071	9.43E-01
Secondary Bile Acid Metabolism	Lipid	12	4	148	-0.069	9.45E-01
Purine Metabolism, (Hypo)Xanthine/Inosine containing	Nucleotide	6	2	148	-0.048	9.62E-01
TCA Cycle	Energy	6	2	148	-0.048	9.62E-01
Acetylated Peptides	Peptide	3	1	148	-0.034	9.73E-01
Dipeptide	Peptide	3	1	148	-0.034	9.73E-01
Fatty Acid Metabolism (also BCAA Metabolism)	Lipid	3	1	148	-0.034	9.73E-01
Glycolysis, Gluconeogenesis, and Pyruvate Metabolism	Carbohydrate	3	1	148	-0.034	9.73E-01
Medium Chain Fatty Acid	Lipid	3	1	148	-0.034	9.73E-01
Nicotinate and Nicotinamide Metabolism	Cofactors and Vitamins	3	1	148	-0.034	9.73E-01
Phenylalanine Metabolism	Amino Acid	3	1	148	-0.034	9.73E-01
Purine Metabolism, Adenine containing	Nucleotide	3	1	148	-0.034	9.73E-01
Androgenic Steroids	Lipid	17	0	148	-3.033	2.42E-03
Ascorbate and Aldarate Metabolism	Cofactors and Vitamins	3	0	148	-1.253	2.10E-01
Diacylglycerol	Lipid	15	0	148	-2.842	4.48E-03

Dihydrosphingomyelins	Lipid	3	0	148	-1.253	2.10E-01
Fatty Acid Metabolism (Acyl Choline)	Lipid	3	0	148	-1.253	2.10E-01
Phosphatidylinositol (PI)	Lipid	4	0	148	-1.449	1.47E-01
Primary Bile Acid Metabolism	Lipid	8	0	148	-2.059	3.95E-02
Progestin Steroids	Lipid	4	0	148	-1.449	1.47E-01

[1]Number of metabolites are the number of detected metabolites assigned to the respective pathway.

Table S4. Pathway enrichment analysis for common metabolites that are associated with both SSB intake and BMI

Sub Pathway	Super Pathway	No. of metabolites assigined to the pathway	No. of significant metabolites in each pathway	Total No. of significant metabolites	Z score	P value
Lysophospholipid (LPL)	Lipid	19	6	28	**4.539**	**5.65E-06**
Phosphatidylcholine (PC)	Lipid	12	4	28	**3.827**	**1.30E-04**
Aminosugar Metabolism	Carbohydrate	3	1	28	1.893	0.0583
Fatty Acid Metabolism (also BCAA Metabolism)	Lipid	3	1	28	1.893	0.0583
Glutathione Metabolism	Amino Acid	3	1	28	1.893	0.0583
Glycolysis, Gluconeogenesis, and Pyruvate Metabolism	Carbohydrate	3	1	28	1.893	0.0583
Sterol	Lipid	3	1	28	1.893	0.0583
Tocopherol Metabolism	Cofactors and Vitamins	4	1	28	1.510	0.1311
Leucine, Isoleucine and Valine Metabolism	Amino Acid	20	0	28	-1.204	0.2285
Benzoate Metabolism	Xenobiotics	19	0	28	-1.172	0.2411
Sphingomyelins	Lipid	18	0	28	-1.140	0.2544
Androgenic Steroids	Lipid	17	0	28	-1.106	0.2686
Fatty Acid Metabolism(Acyl Carnitine)	Lipid	16	0	28	-1.072	0.2838
Diacylglycerol	Lipid	15	0	28	-1.037	0.2999
TCA Cycle	Energy	6	1	28	1.019	0.3081
Tyrosine Metabolism	Amino Acid	14	0	28	-1.000	0.3172
Xanthine Metabolism	Xenobiotics	14	0	28	-1.000	0.3172
Long Chain Fatty Acid	Lipid	12	0	28	-0.924	0.3556

Polyunsaturated Fatty Acid (n3 and n6)	Lipid	12	0	28	-0.924	0.3556
Secondary Bile Acid Metabolism	Lipid	12	0	28	-0.924	0.3556
Urea cycle; Arginine and Proline Metabolism	Amino Acid	12	0	28	-0.924	0.3556
Methionine, Cysteine, SAM and Taurine Metabolism	Amino Acid	11	0	28	-0.883	0.3770
Tryptophan Metabolism	Amino Acid	11	0	28	-0.883	0.3770
Fatty Acid, Monohydroxy	Lipid	10	0	28	-0.841	0.4002
Gamma-glutamyl Amino Acid	Peptide	10	0	28	-0.841	0.4002
Plasmalogen	Lipid	10	0	28	-0.841	0.4002
Fatty Acid, Dicarboxylate	Lipid	9	0	28	-0.797	0.4253
Glutamate Metabolism	Amino Acid	9	0	28	-0.797	0.4253
Histidine Metabolism	Amino Acid	9	0	28	-0.797	0.4253
Lysine Metabolism	Amino Acid	9	0	28	-0.797	0.4253
Monoacylglycerol	Lipid	9	0	28	-0.797	0.4253
Glycine, Serine and Threonine Metabolism	Amino Acid	8	0	28	-0.751	0.4528
Primary Bile Acid Metabolism	Lipid	8	0	28	-0.751	0.4528
Phosphatidylethanolamine (PE)	Lipid	6	0	28	-0.649	0.5166
Purine Metabolism, (Hypo)Xanthine/Inosine containing	Nucleotide	6	0	28	-0.649	0.5166
Pyrimidine Metabolism, Uracil containing	Nucleotide	6	0	28	-0.649	0.5166
Phospholipid Metabolism	Lipid	5	0	28	-0.591	0.5542
Pregnenolone Steroids	Lipid	5	0	28	-0.591	0.5542
Alanine and Aspartate Metabolism	Amino Acid	4	0	28	-0.528	0.5972
Fructose, Mannose and Galactose Metabolism	Carbohydrate	4	0	28	-0.528	0.5972

Lysoplasmalogen	Lipid	4	0	28	-0.528	0.5972
Pentose Metabolism	Carbohydrate	4	0	28	-0.528	0.5972
Phosphatidylinositol (PI)	Lipid	4	0	28	-0.528	0.5972
Progestin Steroids	Lipid	4	0	28	-0.528	0.5972
Acetylated Peptides	Peptide	3	0	28	-0.457	0.6476
Ascorbate and Aldarate Metabolism	Cofactors and Vitamins	3	0	28	-0.457	0.6476
Dihydrosphingomyelins	Lipid	3	0	28	-0.457	0.6476
Dipeptide	Peptide	3	0	28	-0.457	0.6476
Fatty Acid Metabolism (Acyl Choline)	Lipid	3	0	28	-0.457	0.6476
Medium Chain Fatty Acid	Lipid	3	0	28	-0.457	0.6476
Nicotinate and Nicotinamide Metabolism	Cofactors and Vitamins	3	0	28	-0.457	0.6476
Phenylalanine Metabolism	Amino Acid	3	0	28	-0.457	0.6476
Purine Metabolism, Adenine containing	Nucleotide	3	0	28	-0.457	0.6476
Tobacco Metabolite	Xenobiotics	3	0	28	-0.457	0.6476

[1]Number of metabolites are the number of detected metabolites assigned to the respective pathway.
